# Role of community‐based research in advocating HCV prevention and care

**DOI:** 10.1002/jia2.25005

**Published:** 2017-10-04

**Authors:** Patrizia Carrieri, Perrine Roux

**Affiliations:** ^1^ Sciences Economiques & Sociales de la Santé & Traitement de l'Information Médicale INSERM, IRD, SESSTIM Aix Marseille Univ Marseille France; ^2^ ORS PACA Observatoire régional de la santé Provence‐Alpes‐Côte d'Azur Marseille France

**Keywords:** community, stigma, prevention, drug use, hepatitis, harm reduction

In their article, Grebely *et al* 1 clearly present how criminalization and stigma are still the main barriers to hepatitis C virus (HCV) prevention and care for people who inject drugs (PWID). The authors provide perspectives and ways forward to overcome these barriers. They also suggest that taskshifting prevention and care for these groups to community services can be more effective and improve the HCV cascade, as these measures attract most‐at‐risk populations, thanks to reduced stigma.

However, the authors do not mention another important and effective approach: community‐based research. This approach can help to reduce stigma, advocate equity in healthcare and promote change in health policies.

The basic principles of community‐based research are well known. The first is doing *with* people, not *for* people. This means that research is based on the mobilization of concerned groups utilizing a bottom‐up approach, and relies on the expertise of persons living with the disease.

Communities have the power to promote policy and social change. Community‐based research is very effective as its arguments to endorse social transformations are based on real‐world evidence.

There are several important examples of how community‐based research has contributed to change health policy in France. These include ANRS DRAG, which promoted community‐based testing as a tool to attract most‐at‐risk groups 2, ANRS Ipergay, which showed the effectiveness of on‐demand HIV pre‐exposure prophylaxis on HIV incidence in MSM 3, and ANRS AERLI, which evaluated the effectiveness of an existing educational intervention entitled AERLI for HIV and HCV prevention in PWID 4, 5.

More specifically, ANRS AERLI was implemented through a collaboration between the French National Institute of Health and Medical research (INSERM) and two community‐based associations AIDES and MDM, to respond to a specific public health need which was to identify and evaluate innovative harm reduction and HCV preventive interventions. One such existing intervention, called AERLI, was community‐based and focused on reducing injection‐related harm. Conducted by peers and other health staff, it was based on two nested phases: the use of a standardized checklist when supervising injection and a tailored intervention to reduce harm which included information about hepatitis C testing and treatment.

The ANRS AERLI study showed a significant reduction in HCV risk practices and an increase in HCV testing in the intervention group 4, 5. The importance of these results led AERLI to be included as a novel prevention intervention in the new 2016 French health law. This intervention will be now scaled up and replicated in several contexts, such as needle exchange programmes, safe‐injecting facilities and outreach programmes. Its effectiveness will also be evaluated in different European countries. Like needle, syringe and opioid maintenance treatment programmes, AERLI will be used as a new entry point for hepatitis testing and treatment.

In conclusion, if we really aim to “leave no one behind” in the field of HCV, then conducting research with affected communities must be adopted as a complementary approach to reduce inequalities in prevention and care, to advocate changes in health policy and more importantly, to act against criminalization and stigma.

## Competing interests

The authors have no competing interests to declare.

## Authors’ contributions

PC wrote the first draft of the letter and revised it until the final version. PR contributed to the revision of the letter until its current version.

## References

[1] Grebely J , Dore GJ , Morin S , Rockstroh JK , Klein MB . Elimination of HCV as a public health concern among people who inject drugs by 2030 ‐ What will it take to get there? J Int AIDS Soc. 2017;20(1):e22146. 10.7448/IAS.20.1.22146 PMC5577699 28782335

[2] Lorente N , Preau M , Vernay‐Vaisse C , Mora M , Blanche J , Otis J , et al. Expanding access to non‐medicalized community‐based rapid testing to men who have sex with men: an urgent HIV prevention intervention (the ANRS‐DRAG study). PLoS ONE. 2013;8(4):e61225. 23613817 10.1371/journal.pone.0061225 PMC3628708

[3] Molina JM , Charreau I , Spire B , Cotte L , Chas J , Capitant C , et al. Efficacy, safety, and effect on sexual behaviour of on‐demand pre‐exposure prophylaxis for HIV in men who have sex with men: an observational cohort study. Lancet HIV. 2017;4(9):e402–e410. 28747274 10.1016/S2352-3018(17)30089-9

[4] Roux P , Le Gall JM , Debrus M , Protopopescu C , Ndiaye K , Demoulin B , et al. Innovative community‐based educational face‐to‐face intervention to reduce HIV, hepatitis C virus and other blood‐borne infectious risks in difficult‐to‐reach people who inject drugs: results from the ANRS‐AERLI intervention study. Addiction. 2016;111(1):94–106. 26234629 10.1111/add.13089

[5] Roux P , Rojas Castro D , Ndiaye K , Debrus M , Protopopescu C , Le Gall JM , et al. Increased uptake of HCV testing through a community‐based educational intervention in difficult‐to‐reach people who inject drugs: results from the ANRS‐AERLI study. PLoS ONE. 2016;11(6):e0157062. 27294271 10.1371/journal.pone.0157062 PMC4905684

